# Block Copolymer of Flexible and Semi-Flexible Block Confined in Nanopost Array

**DOI:** 10.3390/polym10121301

**Published:** 2018-11-23

**Authors:** Lucia Rišpanová, Zuzana Benková, Peter Cifra

**Affiliations:** 1 Polymer Institute, Slovak Academy of Sciences, Dúbravská cesta 9, 845 41 Bratislava, Slovakia; lrispanova@gmail.com (L.R.), upolzben@savba.sk (Z.B.); 2 LAQV@REQUIMTE, Department of Chemistry and Biochemistry, Faculty of Sciences, University of Porto, Rua do Campo Alegre 687, 4168-007 Porto, Portugal

**Keywords:** block copolymer, semi-flexible macromolecules, molecular simulation, confinement, nanopost arrays

## Abstract

Coarse-grained molecular dynamics simulations of a diblock copolymer consisting of a flexible and semi-flexible block in a dense array of parallel nanoposts with a square lattice packing were performed. The mutual interactions between the two blocks of the confined diblock chain were investigated through a comparison of their size, structure, and penetration among nanoposts with the corresponding separate chains. The geometry of a nanopost array was varied at constant post separation or at constant width of the passage between nanoposts. The size of a single interstitial volume was comparable to or smaller than the size of the diblock chain. A comparison of the blocks with their separate analogous chains revealed that the mutual interactions between the blocks were shielded by the nanoposts and, thus, the blocks behaved independently. At constant passage width, competitive effects of the axial chain extension in interstitial volumes and the lateral chain expansion among interstitial volumes led to a nonmonotonic behavior of the axial span. The position of the maximum in the span plotted against the filling fraction for a diblock chain was dictated by the semi-flexible block. The semi-flexible block penetrates among the nanoposts more readily and the expansion of the whole diblock copolymer is governed by the semiflexible block. The main findings were explained using the free energy arguments when an interstitial volume was approximated by a channel geometry and a passage aperture by a slit geometry. Detail knowledge of controlled conformational behavior in a compartmentalized environment can contribute to new processes in the storage and retrieval of information.

## 1. Introduction

Confinement of macromolecules is a phenomenon frequently occurring in nature and is encountered in many technological applications such as gel electrophoresis [1], partitioning in size-exclusion chromatography [2], etc. Translocation of macromolecules through nanopores also plays an important role in many processes and applications [3]. Recently, scientific attention to the confinement has shifted to more complex confinement topologies. Recent advances in nanofabrication [4] have resulted in preparation of arrays of nanoposts that can be used for DNA separations or as templates for the block copolymer self-assemblies. A nanopost array appears as a possible replacement of a random gel matrix in gel electrophoresis [5,6]. The ability to control the polymer conformation in such regular nanostructures has led to controlled storage or retrieval of genetic information [7], the idea that stems from a comparison to complex natural organization of a DNA in the chromatin.

Detail studies of macromolecular confinements are performed in spherical cavities, in slits, and mostly in nanochannels. Readers are referred to the recent reviews [8,9] for the studies of most often employed nanofluidic channel devices. A dense array of nanoposts investigated here, however, represents a combination of a slit-like and a channel-like confinement, as discussed below. Moreover, this is a network of confinements, which may allow chain partitioning of a macromolecule among many compartments when the size of a macromolecule exceeds the confining interstitial volume. Partitioning of a chain in a confining system of nanopits (entropic traps formed in a nanoslit with a lattice of embedded nanocavities) [10,11] belongs to the related research realm.

Earlier works focused on confinement and partitioning of macromolecules in nanopost arrays considered flexible chains and pointed out an interesting complex non-monotonic behavior of a chain [12]. Subsequently, studies on more complex systems in nanopost arrays were performed on the histone-complexed DNA in an array of nanoposts [7] or on the influence of chain stiffness [13,14]. The studies of the structural behavior of semi-flexible chains at stronger confinement, i.e., in the array of nanoposts with increased crowding by nanoposts, have shown that the stiffer chains penetrate between nanoposts more readily [13,14]. This must have an interesting impact on the behavior of a block copolymer consisting of a flexible and semi-flexible chain, poly(flexible)-*block*-poly(semi-flexible), as a result of an interplay between the topology of connected different blocks and the geometry of nanopost array. Coexistence of flexible and semi-flexible block fragments within a single chain occurs in the transcription process or in other genomic processes since it is known that the single-stranded DNA [15] is much more flexible than the double-stranded DNA. More general interest in the block copolymers comes from nanotechnology, where the block copolymers are especially important in the self-assembly of block copolymers. The self-assembly can also be templated by nanoposts. However, even a single macromolecule with different blocks may bring about interesting effects due to the interaction with its environment. Intriguing is also the tadpole-like conformation of a diblock copolymer composed of blocks that substantially differ in stiffness. The head is formed by relatively compact coil of the flexible copolymer block and the tail is formed by the stiffer copolymer block.

The aim of present work is to characterize the confinement effects of the nanopost array on the conformation of a copolymer chain comprised of a flexible and semi-flexible block. This work is motivated by the previous study [13] of a linear flexible and semi-flexible chain confined in the array of nanoposts where the chain extension as a function of confinement strength displayed a non-monotonic behavior and the peak position was affected by the chain stiffness. We compare this situation to the confinement in a slit and in a channel and explain the observed shift and the non-monotonic behavior of a semi-flexible chain compared to a flexible chain, that has been as yet only observed. Thus, the comparison of the structural behavior of a flexible and semi-flexible homopolymer chain in the nanopost array with a diblock copolymer built up of a flexible and semi-flexible block is the main interest.

## 2. Model and Simulation Method

Using coarse-grained molecular dynamics (MD) the present study concentrates on the structural behavior of a symmetric diblock copolymer consisting of a flexible and semi-flexible block in an array of nanoposts. The structural properties of the whole diblock copolymer as well as of the individual blocks are investigated. The linear symmetric diblock copolymer consists of two blocks of different stiffness containing 1000 beads altogether. The first 499 bonds belong to the flexible block and the second block of 499 bonds is semi-flexible. As the reference and for the comparative purposes a flexible and semilfexible linear chains of 500 beads confined in the nanopost array are also considered. The semi-flexible block of the diblock chain has been represented by discretized bead-spring WLC (wormlike chain) model which has also been used for the flexible part considering zero energy penalty due to deformation of the effective bond angles. Both parts of the copolymer consist of effective monomers (beads) connected by effective fluctuating bonds (springs). The atomistic details are neglected in this model; each bead in the chain is considered to embody effectively several atoms. The beads bear no charge, thus, no component of electrostatic interactions in the total potential energy is considered. The total energy of the system contains contributions due to the bond stretching, the bending of two consecutive effective bonds, and the monomer–monomer and monomer–post pair interactions.

The fully repulsive Weeks–Chandler–Anderson (WCA) represented interactions between the non-bonded as well as bonded effective monomers, where the interaction energy between the beads and the bead diameter was defined by the Lennard–Jones parameters *ε* = 1 and *σ* = 1 and *r_ij_* was the distance between bead *i* and *j*. Throughout this study, the Lennard–Jones *ε* and *σ* parameters are assumed as the units of the energy and distance, respectively.

(1) $$U_{\mathrm{WCA}}\left(r_{ij}\right)=4\varepsilon \left[{\left(\frac{\sigma }{r_{ij}}\right)}^{12}-{\left(\frac{\sigma }{r_{ij}}\right)}^{6}\right]+\varepsilon$$

**Figure 1:**
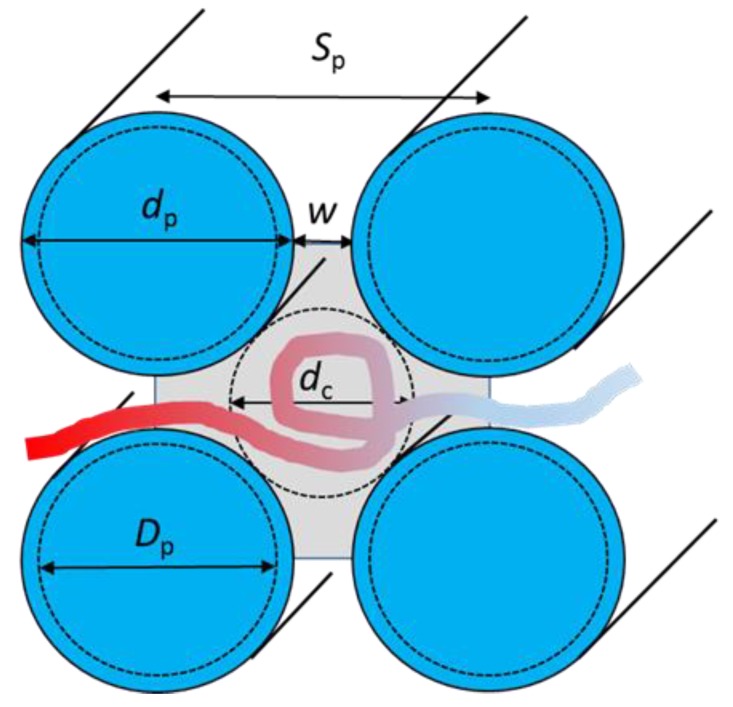
Scheme of the cross-sectional view through four adjacent nanoposts defining the parameters described in the main text.

The attractive part of the WCA potential was removed by setting the cut-off distance to $2^{1/6}\sigma$ using $U_{\mathrm{WCA}}\left(r_{ij}\right)=0$ for $r_{ij}\geq 2^{1/6}\sigma$. Likewise, the same kind of potential was used for the interactions between beads and posts,

(2) $$U_{\mathrm{WCA}}\left(r\right)=4\varepsilon \left[{\left(\frac{\sigma }{r-D_{p}/2}\right)}^{12}-{\left(\frac{\sigma }{r-D_{p}/2}\right)}^{6}\right]+\varepsilon$$

where *r* is the distance between the bead center and the post axis and *D*_p_ is the post diameter. The potential is short ranged and the beads interact with a maximum of the four nearest nanoposts. The scheme defining the array of nanoposts and the geometric characteristic parameters is shown in Figure 1.

Interactions between two bonded beads in the chain were defined by a combination of aforementioned WCA potential and finitely extensible non-linear elastic (FENE) potential

(3) $$U_{\mathrm{FENE}}\left(l_{ij}\right)=-\frac{\kappa }{2}R_{o}^{2}\ln\left[1-{\left(\frac{l_{ij}}{R_{o}}\right)}^{2}\right]$$

where $l_{ij}$ stands for the effective bond length, $\kappa =30\varepsilon \sigma^{-2}$ represents the spring constant and the maximal allowable bond length is $R_{o}=1.5\sigma$. With this combination of potentials, the effective bond length becomes *l* ≈ 0.97*σ* while the size of a bead *w*_b_ is approximately 0.9*σ*. Since the size of a bead and the effective bond length are similar (*w*_b_ ≈ *l*), this chain model belongs to the group of touching bead models of a polymer chain.

The bending potential arising from the chain stiffness was derived from the elastic energy for a WLC model in a continuum limit. In this work, the discretized version of this energy was used:

(4) $$U_{b}\left(\theta \right)=\frac{B}{l}\left(1+\cos\theta \right)$$

where *θ* is the valence angle between two consecutive bonds in a chain and *B* is the elastic constant related to the chain persistence length *P* = *B*/*k*_B_*T*. The dimensionless stiffness parameter, *b* = *B*/*lk*_B_*T,* was set to 20 for semi-flexible chains. This value corresponds to the accepted value of the persistence length for a DNA molecule under the high ionic strength conditions (screened electrostatic interactions), when *w*_b_ ≈ 2.5 nm; *P*/*w*_b_ ≈ 50 nm/2.5 nm = 20. Since relaxed torsional interactions in a chain were considered, no torsional potential was employed. 

**Figure 2:**
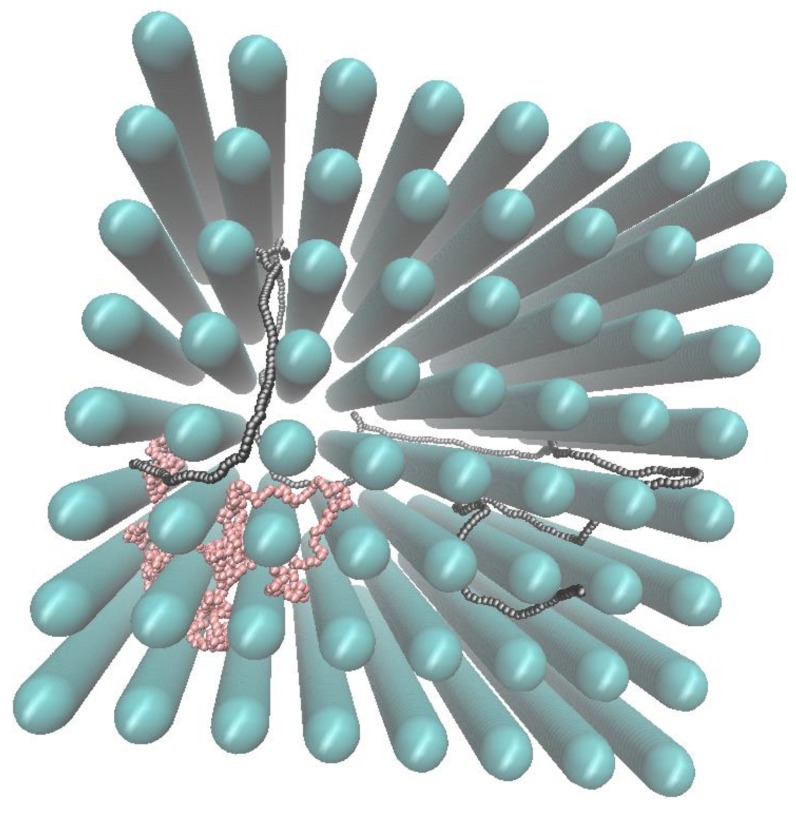
Snapshot of a diblock copolymer composed of the flexible and semi-flexible block in an array of nanoposts with the size *d*_p_ = 6.9 and spacing *S*_p_ = 12. The pink and gray beads belong to the flexible and semi-flexible block, respectively.

The diameter of nanoposts assumed in the simulations is *D*_p_, but the wall of a post interacts with the same repulsive potential as the beads, thus the effective post diameter *d*_p_ becomes broader by the size of the bead, i.e., *d*_p_ = *D*_p_ + 0.9. Then the passage width between posts *w* becomes *w* = *S*_p_ − *d*_p_ and the diameter of an effective interstitial volume approximated as a quasi-channel becomes *d*_c_ = √2*S*_p_ – *d*_p_. The array of collinear posts represented in Figure 1 and Figure 2 was organized in a square lattice geometry, with the characteristic parameters shown in Figure 1. The post axes were aligned with the *x* coordinate. The geometry of the nanopost array is controlled by the distance between posts *S*_p_ or the post diameter *d*_p_. The behavior of a confined polymer, however, results from the confinement strength experienced by a chain among the posts which is characterized by two parameters: the size of interstitial space *d*_c_ and the passage width *w*, the distance between the effective walls of two neighboring posts. The conformations adopted by a confined chain may be considered as a function of the volume fraction of the posts defined as $F=\pi d_{p}^{2}/4S_{p}^{2}$, characterizing the extent of crowding by posts, or as a function of the confinement ratio $d_{c}/w$, characterizing the interplay between the quasi-slit and quasi-channel confinements that govern the partitioning of a chain in the post array. Two types of modification of the nanopost array geometry were assumed. In the first case, the spacing between posts was kept constant at the value *S*_p_ = 12 and the posts diameter *d*_p_ varied in the range of 1.9–11.9, together with narrowing of the passage width. In the second case, the passage width was kept constant at the value *w* = 2 and the spacing between posts *S*_p_ along with the post diameter *d*_p_ varied in the interval 3.9–62.9 and 1.9–60.9, respectively. The same arrangement of the array of collinear nanoposts and the same variations scenario were used in the study of confined linear and circular homopolymer chains [13,14]. A chain with its backbone completely straightened was chosen as the initial conformation. Two initial chain arrangements were considered; one with the chain backbone oriented parallel to the post axes and the second one with the chain backbone oriented perpendicular to the post axes. Both initial conformations provided essentially the same averages of equilibrated properties even under strong confinement. This is one of the criteria suggesting that the equilibration periods of the investigated systems have been sufficiently long.

The MD simulations were performed using the DL Poly Classic package (STFC Daresbury Laboratory, Daresbury, UK) [16] in an *NVT* (constant number of particles, volume and temperature) ensemble. The temperature was kept constant at *T* = *ε*/*k*_B_ applying the Nosé–Hoover thermostat with a relaxation time of 0.1*τ*. The time unit was expressed in the simulation units as *τ* = *σ*(*m*_o_/*ε*)^1/2^, with *m*_o_ = 1 being the unit of mass, and the time step was set to 0.005*τ*. The leap-frog algorithm was implemented for the numerical integration of the chain trajectory. After initial thermal pre-equilibration lasting 10^5^*τ*, the system was equilibrated at *T* = 1*ε*/*k*_B_ for 5.0 × 10^7^*τ.* During the equilibration, the satisfactory oscillations of the potential energy and radius of gyration were achieved. The equilibration was followed by the production phase which lasted for 2.0 × 10^8^*τ*. For the analyses of the structural quantities, 2 × 10^4^ conformations were considered and, the frames were collected every 10,000th step. For each system, the resulting properties were obtained as the average over the all collected frames from three independent simulations.

The structural behavior of the chain confined in the array of nanopost was characterized by the chain size, asymmetry of the chain dimensions, partitioning of the chain monomers among the interstitial volumes and the structure of a polymer chain. The size of the chain was described by the mean span of the chain along the post axes, $R_{s}\equiv \langle \max\left(x_{i}\right)-\min(x_{i})\rangle$, where i∈[1,N], *N* stands for the total number of monomers in a polymer chain and *x*_i_ is the *x*-coordinate of the *i*-th monomer. For a stretched chain in the strong confinement, the mean span approaches the end-to-end distance. The axial span is a main observable factor in single molecule experiments with DNA in nanofluidic devices. The asymmetry of the chain dimensions was studied by the mean square radius of gyration, and its components parallel and perpendicular to the nanoposts, i.e., $R_{g}={\left(\langle R_{g,∥}^{2}\rangle +2\langle R_{g,⊥}^{2}\rangle \right)}^{1/2}$, $R_{g,∥}={\left(<R_{g,x}^{2}>\right)}^{1/2}$, and $R_{g,⊥}={\left[(<R_{g,y}^{2}>+<R_{g,z}^{2}>)/2\right]}^{1/2}$, respectively. The organization of the monomers on different length scales depending on the wave vector *q* = 2*π/Ω* was investigated by the structure factor *S*(*q*) of the diblock chain as well as of the individual blocks. Here, *Ω* represented the length scale ranging from the monomer size up to about the size of chain. The structure factor is defined as follows:

(5) $$S\left(q\right)=\frac{1}{N^{2}}\langle \overset{N}{\underset{i=1}{\sum }}\overset{N}{\underset{j=1}{\sum }}\sin\left(qr_{ij}\right)/qr_{ij}\rangle$$

where *r*_ij_ is the distance between chain segments *i* and *j*. The extent of lateral partitioning of the chain monomers among the interstitial volumes in the array of nanoposts was quantified by the occupation number *n*, the average number of interstitial spaces occupied by the polymer chain. One interstitial space is indicated in Figure 1 by a shaded area.

## 3. Results and Discussion

The properties of a diblock copolymer in the array of nanoposts are investigated under two different variations of the array geometry: 1) alteration of the post size *d*_p_ at the constant distance between posts *S*_p_ (accompanied by the respective variation of the passage width *w*); and 2) correlated variation of *d*_p_ and *S*_p_ at the constant passage width *w*. The typical structure of the diblock copolymer in a nanopost array from the simulations is shown in Figure 2.

### 3.1. Crowding by Nanoposts at Constant Post Spacing S_p_

**Figure 3:**
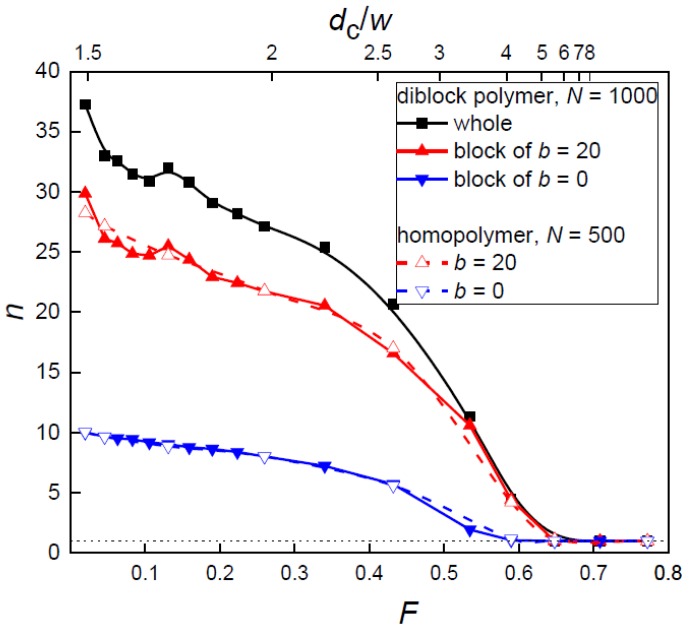
Occupation number *n* of a diblock chain, its blocks and homopolymer chains corresponding to the blocks as a function of the filling fraction of posts *F* or the confinement ratio *d*_c_/*w* for constant post separation *S*_p_ = 12 and varying *d*_p_. Values of *d*_p_ are presented in the corresponding Figure 4.

First, the filling fraction of the posts *F* in the system was increased at the constant posts separation, which means that the post diameter was increased at the fixed separation between the nanoposts. It is instructive at this point to realize that before the posts touch each other (*D*_p_ = *S*_p_) at the maximum filling fraction, *F*_max_ = π/4 = 0.7854, there is another limit at which the passage width *w* becomes equal to the bead size *σ* and the chain cannot penetrate between the interstitial volumes. This most narrow allowable passage occurs for *S*_p_ = 12 at *d*_p_ = 11.1 according to $w=S_{p}-d_{p}$ which corresponds to the filling fraction *F* = 0.672 or confinement ratio *d*_c_/*w* = 6.52. Accordingly, in the plot of occupancy of interstitial volumes at these values only single occupancy of interstitial volumes occurs in both systems of flexible and semi-flexible chain (Figure 3). 

Interestingly, the onset of the lateral spreading of the chains in the nanopost array, i.e., the penetration transition from a single to multiple occupancies of interstitial volumes depends on the chain stiffness. The penetration transition is identified with the *d*_c_/*w* value at which it exceeds 1. It occurs at *F* = 0.59 and 0.65 (*d*_c_/*w* = 4.1 and 5.5) for the flexible and semi-flexible block as well as for the respective individual homopolymer chains, respectively. This indicates that in comparison with the flexible block, the stiffer block spreads more readily among the nanoposts at the same conditions and needs stronger confinement in order to become restricted into a single interstitial volume. Apart from the investigation of the whole block copolymer, the behavior of its individual blocks was also analyzed. It is clear that the overall value of the occupation number *n* stems mainly from the spreading of the semi-flexible block, while the contribution from the flexible block represents only a minor contribution. The position of the spreading transition of the whole diblock is predominantly dictated by the stiffer block as well. In Figure 3, the occupancy by the individual blocks is also compared to the equivalent homopolymer chains. Over the whole range of crowding by the posts *F*, these two occupation curves are nearly identical. This is attributed to the absence of the interactions between the two blocks in the diblock copolymer as they are spread among the nanoposts and, thus, are mutually separated. This will be analyzed and interpreted also for the variation of the array geometry at the constant passage width *w*, and will be compared to an unconfined diblock chain. The statistical uncertainties are more significant for the stiffer chain or block; however, it does not account to more than 5%.

It should be also noted that the increase of occupation number with the decrease of the filling fraction *F* reflects the decrease of confinement, which is in line with the classical partitioning behavior of polymer chains that partition readily to a weaker confinement strength. This is important to mention since the situation in the next system, for the constant passage width *w*, will be more complex.

**Figure 4:**
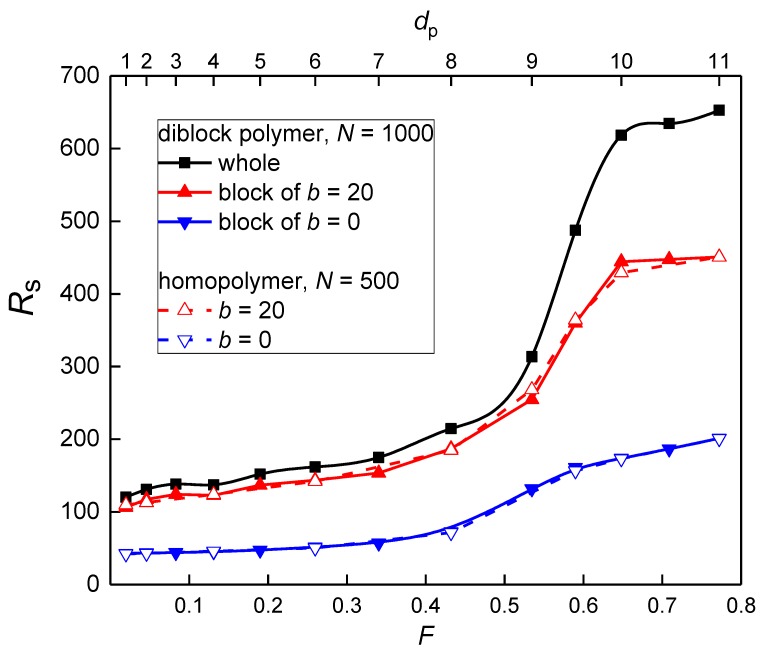
The axial span of a diblock chain, its blocks and homopolymer chains corresponding to the blocks as a function of the filling fraction of posts *F* or nanopost diameter *d*_p_ for constant post separation *S*_p_ = 12.

The span behavior of the diblock copolymer in the nanopost array, depicted in Figure 4, represents the axial elongation of the copolymer in the quasi-cylindrical interstitial volumes and reflects also the lateral penetration transition quantified by the occupation number *n*, shown above in (Figure 3). The overall span monotonically increases and levels off at high *F* where the diblock chain is stretched along the posts in a single narrow interstitial volume. As the post diameter decreases the interstitial volumes become broader and the chain penetrates into more interstitial volumes. In broader interstitial volumes, a chain has more space to relax in the lateral directions and at the same time, there is also a lateral expansion of a chain over more interstitial volumes. The decrease of span comes also from the fact that on penetration to neighboring interstitial channel-like volumes the chain may follow an opposite direction along the posts. As with the occupation number, the span and the observed transition are mainly governed by the stiffer block. The span of the individual blocks is additive only at a high filling fraction, *F* ≥ 0.65, where the whole chain resides in a single interstitial volume. At lower *F*, the additivity does not hold. As in the case of the occupation number, the span of the individual blocks does not differ from the span of the individual homopolymer chain over the whole range of *F*.

**Figure 5:**
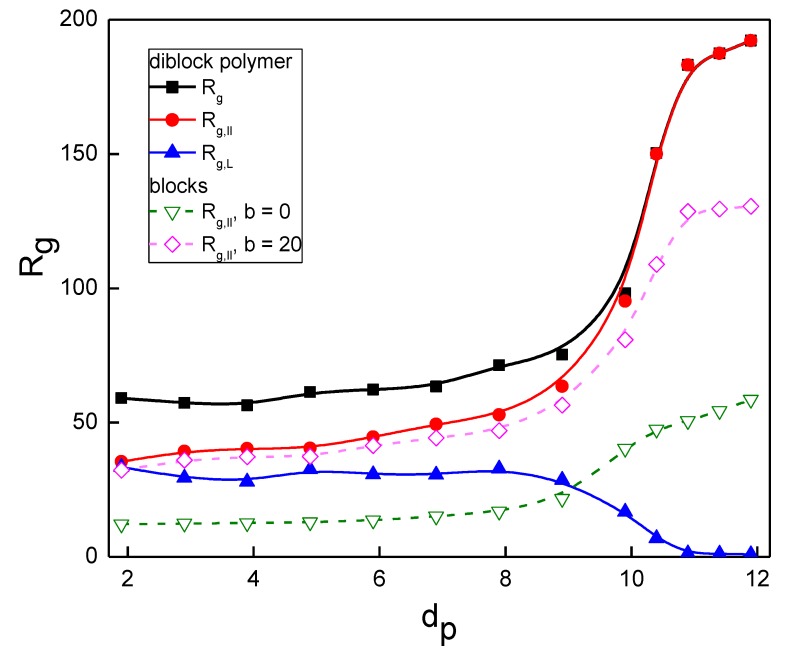
The radius of gyration of a diblock copolymer and its components parallel and perpendicular with the nanoposts. The parallel component of the radius of gyration for individual blocks is shown by the dashed lines.

The radius of gyration *R*_g_ of the block copolymer in the nanopost array and its parallel and perpendicular components with respect to the nanopost axes,$\;R_{g,∥}$ and $R_{g,⊥}$, have also been estimated. These quantities provide an insight into the chain asymmetry induced by the nanopost array and in one property provide a picture that is related to the extension along posts *R*_s_ together with the lateral spreading *n*, the properties which were investigated separately above. While$\;R_{g,∥}$ of the diblock chain increases with the increasing posts size *d*_p_ (increasing nanopost crowding *F*) and tends to a plateau given by the maximum axial chain extension for the geometry of array, the perpendicular component $R_{g,⊥}$ substantially decreases in this region, as seen in Figure 5 for the same systems as in Figure 3 and Figure 4. In addition to the diblock copolymer, the parallel component $R_{g,∥}$ is also shown for individual blocks. The additivity of $R_{g,∥}$ for the two blocks in the overall $R_{g,∥}$ is valid only for the narrowest interstitial volumes, similar to the situation with the span in Figure 4. In this region of strong confinement, the behavior of both blocks differs, however. While the semi-flexible block tends to plateau, approximately at *d*_p_ = 10.9, the flexible block is still further extending on the increasing confinement strength. A comparison of the radius of gyration of the individual blocks with the respective homopolymer chains in the same nanopost array does not bring a difference, similarly to the span and occupation number (Figure 3 and Figure 4) and is not shown in Figure 5.

### 3.2. Crowding by Nanoposts at Constant Passage Width w

**Figure 6:**
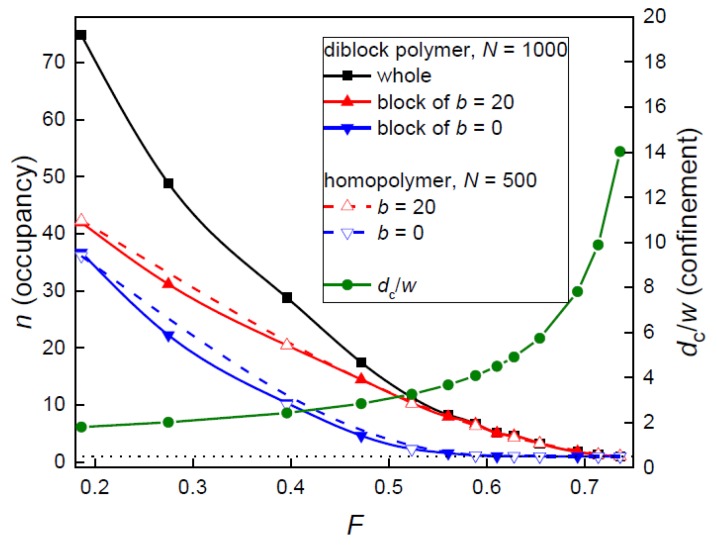
Occupation number *n* of a diblock chain, its blocks and homopolymer chains corresponding to the blocks together with the confinement ratio *d*_c_/*w* as functions of the filling fraction of posts *F* for constant narrow passage width *w* = 2.

The second way of the geometry variation of the nanopost array was realized at the narrow passage width, *w* = 2. In Figure 6, the occupation number of the diblock copolymer and its individual blocks is shown along with the extent of confinement expressed by the ratio *d*_c_/*w* as functions of the filling fraction by nanoposts *F*.

The onset of the multiple occupancies of interstitial volumes occurs at *F* = 0.59 and 0.71 (*d*_c_/*w* = 4.1 and 9.9) for flexible and for the semi-flexible block as well as for the respective individual homopolymer chains, respectively. This shift of the penetration transition is analogous to that observed at the constant post separation investigated in the previous section. The penetration of the whole diblock chain which governs the overall lateral spreading of the diblock chain in the nanopost array follows the penetration of the stiffer block. 

One can also see that the occupation number *n* displays an opposite trend with the confinement ratio *d*_c_/*w*. It should be noticed that the opposite trends in these two properties have also been observed for the geometry variation of the nanopost array at constant post separation (Figure 3). However, at constant *w,* the increase of confinement ratio *d*_c_/*w* is given solely by the increase of *d*_c_. The increased partitioning *n* among more narrow interstitial volumes (characterized by their quasi-diameter *d*_c_) observed here is in contrast with the classical partitioning of polymer chains that is characteristic by easier partitioning to a weaker confinement (or larger *d*_c_) from the bulk solution. The partitioning of a chain in the post array, however, is governed not only by *d*_c_ but also by *w*. Even though the slit width *w* is constant it represents a lower entropic barrier as *d*_c_ decreases, which enables an easier penetration to the neighboring interstitial volumes. In the array of nanoposts, the free energy of a confined chain is reduced by enhanced chain partitioning among the interstitial volumes.

**Figure 7:**
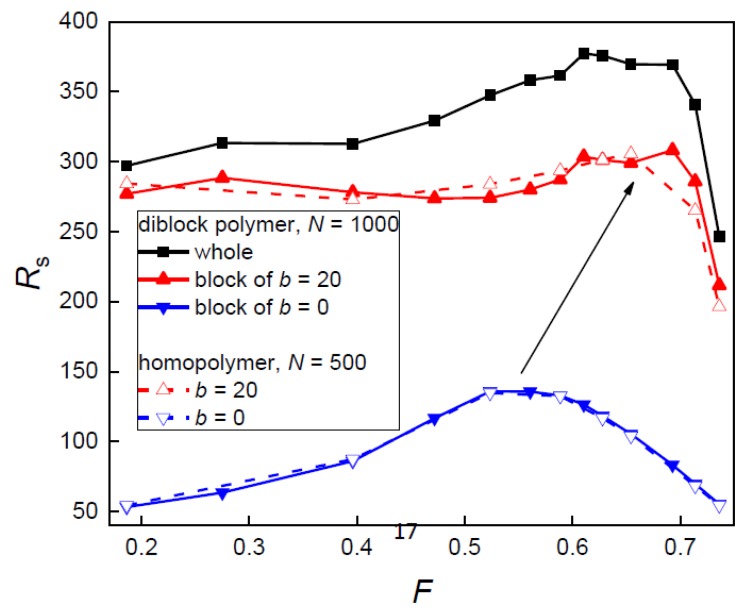
The axial span of a diblock chain, its blocks and homopolymer chains corresponding to the blocks as a function of the filling fraction of posts *F* for constant passage width *w* = 2.

In contrast with the geometry variation at constant post separation, the span of the diblock copolymer along with the nanoposts at narrow constant passage width exhibits interesting maximum, Figure 7, similar to that already reported for homopolymer chains [12,13]. It appears as a result of two opposing tendencies. At high *F*, the diblock chain resides in a single interstitial volume that is sufficiently broad relatively to the passage width *w*. As *F* decreases also the width of interstitial volume *d*_c_ decreases and thus the span increases. At the same time, the more restricted diblock chain starts to penetrate into more interstitial volumes and thus expands in the lateral direction which leads to the axial chain contraction. The diblock chain sequence within narrower individual interstitial volumes becomes more axially stretched. The maximum appears as an interplay of these effects. At high *F* values, the effect of the axial chain extension due to the narrowing of interstitial volumes prevails while at lower *F* values, the effect of the lateral chain expansion, when parts of the chain may follow opposite direction along the post axes, dominates. For the situation of single volume occupancy, at high *F*, the quasi-biaxial confinement approximation of interstitial volumes has been confirmed by investigation of *R*_s_ *vs d*_c_ dependence. This dependence follows $R_{s}\sim d_{c}^{-x}$ with *x* = 2/3 and 1 for the flexible and semi-flexible chain, respectively, in agreement with the exponent values predicted for the evolution of the chain axial expansion with the channel diameter [17]. When compared the semi-flexible block with its free analogue (*N* = 500) one can see the increased uncertainties under strong confinement (larger *F*). This is not surprising since the block is a part of a longer chain (*N* = 1000). In order to reach the same precision as for the shorter chains, these systems would need larger total simulation time to reach the same statistics as the shorter chains. However, this uncertainty does not exceed 10%.

As one can expect from the independent behavior of the two blocks evidenced in the previous discussion, the positions of a maximum for the flexible and semi-flexible block are different in Figure 7. The position of a maximum for the whole diblock chain follows the position for the semi-flexible block. The position of a maximum and its shift with the variation of the chain stiffness is very close to position observed for the transition to a multiple occupancy *n* (Figure 6) and also to the transition positions found for the occupation number and span in the case of constant post separation (Figure 3 and Figure 4). This stiffness dependence of the transition position has been already pointed out for homopolymers [13]. The estimation of this transition is based on the equality of the confinement free energy penalty of a chain experienced in the passage aperture between two neighboring posts and in the interstitial volume among four nearest posts since a chain segment has to cross the passage aperture during its translocation between two neighboring interstitial volumes. In order to express the free energy of a confined chain as a function of the geometrical parameters of the nanopost arrays (Figure 1), the passage aperture may be viewed as a quasi-slit of height *w* and the interstitial volume can be viewed as a quasi-channel of diameter *d*_c_. The expressions for the free energy penalties of a chain confined in a slit and a channel of square cross-section are collected in a recent review [8]. The confinement free energy for a semi-flexible chain in the channel with the square cross-section under strong confinement is given by [18,19]:

*ΔA*/*k*_B_*T* = 2.2072 *L P*^−1/3^*D*^−2/3^ (6)

where *L* is the chain contour length and *D* is the channel width. For a chain under moderate confinement in a square channel, the free energy reads [20]:

*ΔA*/*k*_B_*T* = 4.0 *L* (*Pa*)^1/3^*D*^−5/3^ (7)

For the free energy of a semi-flexible chain in a narrow slit at strong confinement, Equation (6) divided by a factor of 2 was used since the free energy penalty of a chain in a square channel is, to a good approximation, twice that in a slit of the height equal to the channel size [18,19,21]. The representation of the strong confinement induced by a passage aperture using Equation (6) requires *D* to be replaced by *w*. In a similar vein, *D* has been replaced by *d*_c_ for the representation of the moderate confinement induced by an interstitial channel-like volume, using Equation (7). This is only an approximation for what is actually not a perfect but leaky channel (Figure 1) and, thus, the size *d*_c_ represents only the effective channel diameter. Also, the passage aperture can be approximated by the slit geometry only on short length scale near to the passage width *w*. Making equal these two expressions of the free energies one obtains the following condition for the confinement ratio at the transition of semi-flexible chain from a single to multiple occupancies of interstitial volumes in the post array:

*d*_c_/*w* = 2.165 (*P*/*w*)^2/5^(*a*/*w*)^1/5^ (8)

For the persistence lengths of a semi-flexible chain (*b* = 20), *P* = 19.23, (obtained from the orientation correlations of a corresponding free chain), and for the size parameters, *w* = 2, *a* = *l* = 0.97, the respective predicted confinement ratio for the penetration transition becomes *d*_c_/*w* = 4.63. The same procedure has been also employed for the estimation of the penetration transition of a confined flexible chain. However, the conformation of a flexible chain under strong slit-like confinement follows the de Gennes regime (Equation (7)) instead of the Odijk regime (Equation (6)). Thus, Equation (7) has been adopted to estimate also the free energy of a flexible chain in a quasi-slit passage aperture instead of Equation (6). In this case, the expression is less complex and does not depend on the chain stiffness, Equation (9).

*d*_c_/*w* = 2^3/5^= 1.52 (9)

In fact, the confinement ratio is *d*_c_/*w* = 1.52 and does not depend on the geometric parameters of the nanopost array [13,14]. Thus, this value should not depend on how the geometry of the nanopost array is modified. Table 1 compares the *d*_c_/*w* values for the penetration transitions obtained from the simulations with the predicted values. Evidently, the trend of the transition shift with chain flexibility obtained from simulations and prediction based on the free energy arguments is confirmed but is consistent only qualitatively. Worth noting is, however, that the geometry parameters of the nanopost array at which the penetration transition occurs for the flexible chain essentially depend only on the confinement ratio and not on the array geometry consistently with the prediction.

As has been shown, the blocks and the respective homopolymers exhibit almost identical plots of the occupation number and axial span in the whole range of *F*, both at constant *S*_p_ and *w*, (Figure 3, Figure 4, Figure 6 and Figure 7). This means that the blocks in the diblock copolymer are sufficiently separated and do not affect each other in the nanopost array. It is interesting to know if this independence results from the presence of the posts in the array or is present also in the free diblock copolymer. A comparison of the span of the individual blocks constituting the free diblock copolymer with the span of the respective free homopolymers reveals that the blocks in the free diblock chain are slightly more expanded. The span of the flexible block (*b* = 0) in the free block copolymer is 40.06 in comparison to 38.08 of the respective individual free chain, while the semi-flexible block (*b* = 20) in the diblock chain spans to 93.66 in comparison to 91.77 of the respective individual free chain. However, when the block copolymer is placed into the nanopost array the weak excluded volume effect between the two blocks that are visible in the free diblock copolymer are shaded by the nanoposts which separate and compartmentalize the diblock chain. In addition to the hindered interactions between the blocks, the posts lead to a certain extension of the chain along the posts. This is demonstrated when the span values of the blocks for the confined diblock chain presented in Figure 4 are compared with the aforementioned span values for the respective blocks of the free diblock chain. This enhanced span of the confined blocks is preserved even in the array composed of the narrowest investigated posts of *d*_p_ = 1.9. In this weak confinement, the span of the flexible and semi-flexible block is 42.7 and 106.5, respectively.

It should be noted that the non-monotonic behavior of a chain size plotted against the filling fraction was observed also for a flexible chain in a porous medium which was modeled by crowded spherical particles instead of nanoposts [22]. This confinement, however led to a minimum in the chain size *vs* filling fraction dependence in contrast to a maximum observed in this and previous studies. This difference has already been explained [12,13]. Recent studies pointed out that the effect of particles on polymers is generally compressive if the particles are mobile [23], while the situation is more complicated if the positions of the particles are quenched [22].

### 3.3. Structure Factor

**Figure 8:**
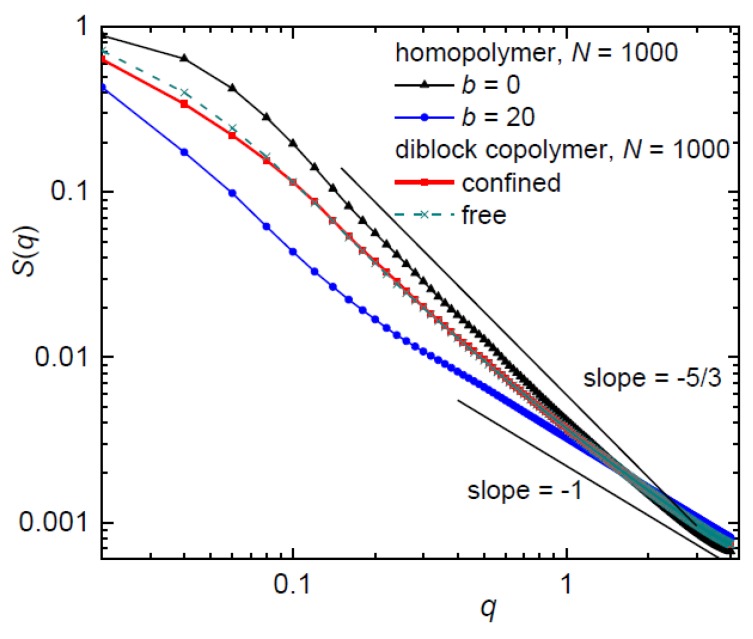
Structure factor for a diblock copolymer and respective flexible and semi-flexible homopolymer in a weakly confining array of *d*_p_ = 1.9, *S*_p_ = 12. A free unconfined diblock copolymer is also shown for comparison. The lines with the slopes −5/3 and −1 represent theoretical predictions for a flexible and semi-flexible/stiff homopolymer chain, respectively.

Since it has been shown that the behavior of a chain only weakly confined in the nanopost array is akin to the behavior of a free chain [13,14] the structure of the weakly confined diblock copolymer for narrow and well-separated posts, *d*_p_ = 1.9, *S*_p_ = 12, is shown in Figure 8. The predicted relation *S*(*q*) ~ *q*^−1/*ν*^ (*ν* is the scaling Flory exponent) gives rise to a dependence of different complexity dictated by the number of different length scales detectable in a chain. *S*(*q*) of the flexible homopolymer is characterized by the slopes −5/3 characterizing the organization of monomers with excluded volume effects and *S*(*q*) of the semi-flexible homopolymer displays the slope −1 characteristic for the rod-like arrangement of monomers at high *q* values corresponding to the persistence length. However, *S*(*q*) of the diblock copolymer is found between *S*(*q*) of the flexible and semi-flexible homopolymer. In consistence with the conformational behavior of the homopolymers [13,14], the diblock copolymer weakly confined in an array of very narrow posts with the separation much larger than the post diameter behaves like the corresponding free diblock copolymer.

**Figure 9:**
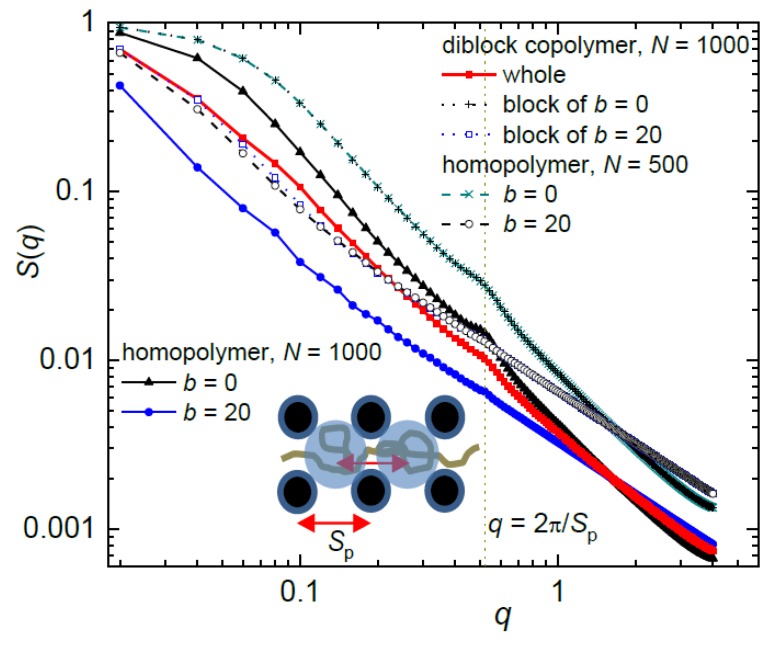
Structure factor of a diblock chain, its blocks and homopolymer chains corresponding to the blocks as well as a flexible and semi-flexible homopolymer of the same length as a copolymer at the moderate confinement of a post array with *d*_p_ = 6.9, *S*_p_ = 12. The dashed line indicates the *q* value corresponding to the distance between the axes of the adjacent interstitial volumes, *S*_p_, outlined in the inset.

The structure factor of the diblock copolymer moderately confined in the array of broader posts with *d*_p_ = 6.9 and *S*_p_ = 12 is presented in Figure 9. In this nanopost array, both block constituents occupy more interstitial volumes and the geometry of the array is reflected in the structure factor of the chain. While the whole diblock chain occupies *n* = 26.7 interstitial volumes, the occupation number of the semi-flexible block is 21.5 and of the flexible block only 8.05. Figure 9 also presents the structure factor for the respective flexible and semi-flexible homopolymer of the same length as the diblock copolymer as well as for the individual blocks within the copolymer along with their respective homopolymers. As for the chain span and occupation number, the structure factor of the blocks within the diblock chain and of the respective separate homopolymers is almost indistinguishable. The presence of the broader posts is demonstrated in the structure factor of the diblock chain and its constituting blocks by a hump positioned at the characteristic value *q* ≈ 2*π*/*S*_p_ as assigned by the vertical line in Figure 9. This hump indicates the enhanced correlations between the chain monomers which occupy two adjacent interstitial volumes separated by *S*_p_. This enhancement of local density is more significant for the flexible chains as well as for the flexible block in the diblock chain which populate the neighboring interstitial volumes more densely than the semi-flexible analogous. Notice also that there are 8 neighbors in the first shell of the interstitial volumes which agrees with the occupation number of the flexible block *n* = 8.05. Since the occupation number of the semi-flexible block is 21.5, fewer monomers are located in the neighboring interstitial volumes when compared with the flexible block. 

**Figure 10:**
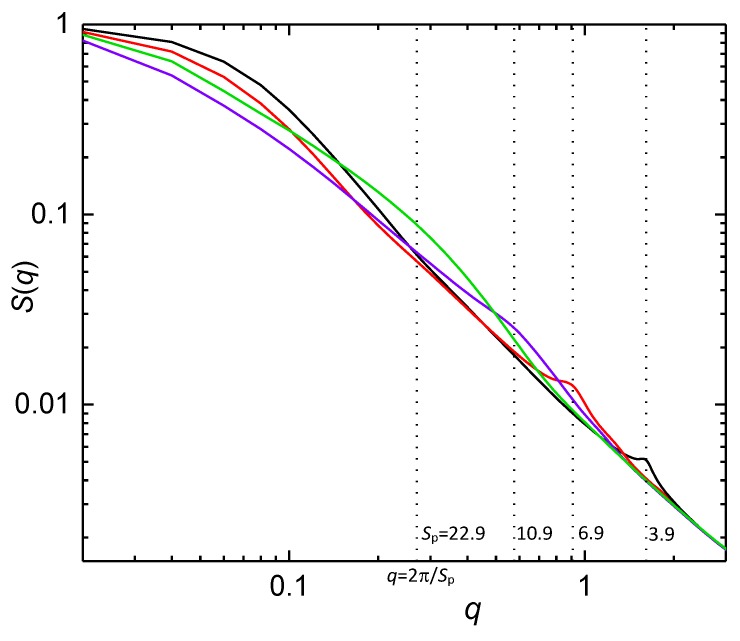
Structure factor for a flexible block of a block copolymer in an array of nanoposts with constant passage width *w* = 2 and variable *S*_p_ and *d*_p_. The vertical dashed lines indicate the increased segment correlations at the length scale *S*_p_, the effective distance between the axes of two adjacent interstitial volumes.

The origin of the hump in the structure factor of a chain confined in the post array can be confirmed by plotting *S*(*q*) for a chain in the post arrays of various post separations. The results of such a variation are presented in Figure 10 for the flexible block of the diblock copolymer situated in four different post arrays with constant *w* = 2 and altering *S*_p_. One can see that the position of the enhanced intensity of the structure factor correlates well with the post separation which is, at the same time, also the distance between the axes of the adjacent interstitial volumes. The hump in the structure factor is also detectable for the diblock copolymer, though, it is less intensive than for the flexible block, but more intensive than for the semi-flexible block (Figure 9).

## 4. Conclusions

This study concerns the conformational behavior of the flexible and semi-flexible chain in the array of nanoposts and the extent to which the behavior of these separate chains is preserved in the diblock copolymer built of two corresponding blocks in the same environment. 

The geometry of the nanopost array has been modified in two ways: at the constant posts separation and at the constant passage width between nanoposts. In the cases of the flexible chain, the onset of the chain penetration from a single to multiple interstitial volumes occurs at the same values of the ratio of the width of interstitial volume and the passage width between the nanoposts in agreement with the predictions based on free energy arguments. The situation is more complex for the semi-flexible chain where the penetration transition depends also on the chain stiffness (persistence length) and the geometry of the post array. 

The occupation number and the axial chain span suggest that the flexible and semi-flexible blocks in the diblock copolymer do not influence each other and their properties are close to the properties of the corresponding individual chains in the arrays. This absence of the interactions between the blocks is observed for both modifications of the array geometry and is attributed to the separation and shielding of the blocks by the nanoposts. The behavior of the whole diblock copolymer is determined by the behavior of the stiffer block.

Two interesting findings have been observed during the chain partitioning among the nanoposts. The chain partitioning is enhanced with the increasing confinement strength, i.e., with broadening of the post which is opposite to the partitioning between a bulk solution and confinement. The partitioning is enhanced for the stiffer chains or blocks. Both phenomena are rationalized in terms of the free energy penalty of a confined chain which has to penetrate through the passage apertures during its partitioning among the interstitial volumes. The interstitial volume and a passage aperture are approximated by a channel and a slit, respectively. The former phenomenon is attributed to the drop in the excess free energy of a chain when it penetrates through the passage apertures into more interstitial volumes. The latter phenomenon is explained by the lower free energy penalty of a stiffer chain in a narrow slit which represents the passage aperture. These findings are in qualitative accordance with the prediction.

When the geometry of the nanopost array is modified at the constant passage width the span of the diblock chain along the nanopost axes exhibits a maximum when plotted against the filling fraction. The position of this maximum is dictated by the position of the maximum for the semi-flexible block. This effect originates from a compromise between two opposing effects accompanying this modification of the array geometry. It results from the chain extension on narrowing the channel of interstitial volumes and from subsequent penetration to neighboring volumes on further narrowing, which in turn results in the span decrease (although the chain is extended more locally in the narrower channel it may follow opposite direction in the neighboring interstitial volumes). The maximum position correlates well with the position of the penetration transition that depends on the chain stiffness.

The structure factor of the investigated chains reflects the organization of monomers within the chains and, for a sufficiently strong confinement, also the geometry of the array. The latter is more pronounced for the flexible chains or flexible block, less pronounced for the diblock copolymer, and marginal for the semi-flexible chains or semi-flexible block. 

In addition to the variation of the array geometry at the constant post separation or constant passage width, the array geometry may be modified at the constant post diameter as well. Currently, such a study is being carried out and the results also show interesting non-monotonic behavior and some novel feature.

## Figures and Tables

**Table 1:** Observed and predicted confinement ratio *d*_c_/*w* at the penetration transition between the interstitial volumes for a flexible and semi-flexible chain. The relative standard deviations are <0.1% and ~9%, respectively, for the flexible and semi-flexible chains in the nanopost arrays of both geometry variations.

	*b* = 0		*b* = 20	
	*d*_c_/*w*	*F*	*d*_c_/*w*	*F*
Constant *S*_p_	4.1	0.59	5.5	0.65
Constant *w*	4.1	0.59	9.9	0.71
predicted	1.52		4.63	
